# The Potential for Testing Stool to Reduce Tuberculosis Missed Diagnoses and Misdiagnoses

**DOI:** 10.4269/ajtmh.18-0507

**Published:** 2018-08

**Authors:** Sumona Datta, Luis E. Gonzales-Huerta, Carlton A. Evans

**Affiliations:** 1 Section of Infectious Diseases and Immunity, Imperial College London, and Wellcome Trust Imperial College Centre for Global Health Research, London, United Kingdom;; 2 Innovation for Health and Development (IFHAD), Laboratory of Research and Development, Universidad Peruana Cayetano Heredia, Lima, Peru;; 3 Innovación Por la Salud Y Desarrollo (IPSYD), Asociación Benéfica PRISMA, Lima, Peru

Tuberculosis kills more people than any other infection, mainly in resource-constrained settings where diagnosing tuberculosis and detecting drug resistance are particularly challenging.^1^ All tuberculosis tests frequently have false-negative results, causing some patients to miss out on the tuberculosis treatment they need, patients with suspected tuberculosis to require multiple tests before diagnosis, delayed diagnosis, and a significant minority of patients to start treatment of tuberculosis empirically, without bacteriological confirmation of diagnosis or assessment of drug susceptibility.^2^ Inevitably, some of the patients receiving empirical tuberculosis therapy do so inappropriately, unnecessarily experiencing stigma, costs, and toxicity, and sometimes causing them to miss out on potentially life-saving therapy for their actual disease process.^3,4^

Difficulties diagnosing tuberculosis and determining drug susceptibility partly result from the inadequacy of appropriate technology tests. Chest radiography has moderate sensitivity and poor specificity. The only laboratory test that most patients have access to globally is smear microscopy, which for more than a century has had such low sensitivity that approximately half of people with tuberculosis have false-negative results.^5^ Culture is the most sensitive test and provides the most clinically reliable assessment of drug sensitivity, but it is technically demanding, potentially hazardous to laboratory personnel, and generally takes weeks to complete.^1^ Polymerase chain reaction (PCR) has great potential because it takes only a few hours, has intermediate sensitivity between smear and culture for sputum analysis, and predicts drug resistance fairly reliably.^1^ However, the need for carefully selected controls in the evaluation of all laboratory diagnostic tests has been largely neglected, which is important because culture and in-house PCR are both prone to false-positive and false-negative results.^6–11^

Poor tuberculosis test performance is confounded by the quality of diagnostic specimens.^12^ Most tuberculosis affects the lungs, for which sputum is the main diagnostic specimen, and strategies to increase the quality of sputum collection have recently been clarified.^12^ However, it is difficult or impossible to collect sputum from some adults and most young children, for whom the relative merits of testing saliva, induced sputum, urine, nasogastric aspirates, or swallowed string are poorly defined.^13,14^ Most sputum is swallowed, and both tuberculosis bacteria and their DNA survive passage through the intestinal tract, so stool samples may be tested for the evidence of pulmonary tuberculosis and drug susceptibility.^15–17^ However, the feasibility of using stool to diagnose pulmonary tuberculosis is limited by the observations that swallowed sputum in stool is likely to be considerably diluted within the intestinal tract, culture-based detection may be impaired by gastric acid killing mycobacteria, stool flora can contaminate cultures, and stool may contain PCR inhibitors.^17^

In this issue of The *American Journal of Tropical Medicine and Hygiene*, Andrew DiNardo et al.^6^ report an important advance in this field. They adapted a DNA extraction and concentration technique developed for soil samples with an in-house PCR technique to study detection of mycobacteria in stool. This considerably improved the limit of the detection of *Mycobacterium tuberculosis* DNA in stool to be comparable with that of the most widely used techniques for sputum. Indeed, in patients with sputum culture–positive tuberculosis, stool PCR was considerably more sensitive than sputum microscopy—a remarkable finding. These results are comparable with those from pilot studies in pediatric populations in which PCR was used to compare small-volume stool samples with induced sputum samples.^18^ Concordant with these findings, in HIV-coinfected children, PCR testing was shown to have similar diagnostic yield for stool, expectorated sputum, and gastric aspirates.^19^ If validated, the novel sample processing approach described by DiNardo et al. may be transformative for diagnosing tuberculosis by stool PCR and perhaps also by PCR of other specimens. This is important because the highest risk patients with tuberculosis such as children and people living with human immunodeficiency virus usually have paucibacillary sputum, which urgently requires new techniques for concentrating mycobacterial DNA. Currently, centrifugation is the main technique used for concentrating mycobacteria in clinical specimens, but this is inefficient and biohazardous, and alternatives such as filtration or flotation have so far been problematic.^20^ Although this study offers a significant advance, some aspects of the methodology will require refinement before progressing from proof-of-concept to implementation. It is also noteworthy that most of the few participants who were unable to produce sputum or had presumed false-negative sputum tuberculosis test results were also negative on stool testing. Indeed, even if a new test had 100% sensitivity and specificity for tuberculosis, this would be hard to demonstrate in people with suspected sputum-scarce or paucibacillary tuberculosis because in these groups definitively proving the presence or absence of tuberculosis is often impossible.

The tuberculosis diagnostic landscape is advancing rapidly but tools to monitor treatment response are relatively neglected. Monitoring treatment response in patients with pulmonary tuberculosis provides information about patient clinical status, has the potential to predict and enable prevention of treatment failure, and also guides infection control. The tool most commonly used to monitor treatment response is sputum-smear microscopy, which relies on the patient producing good quality sputum, and is generally only useful for patients who were smear-positive at the start of treatment. DiNardo et al. report that patients who remained stool PCR positive after 2 months of therapy were 2.8 times more likely to have drug resistance or treatment failure, although sensitivity was only 44%.^6^ Poor treatment response has multiple causes, but the most common is inadequate treatment of unrecognized drug-resistant tuberculosis. Therefore, rapid drug susceptibility testing should be provided to all patients at the start of treatment. Even when this policy is optimally implemented, tuberculosis treatment sometimes fails. Changes in symptoms, body weight, and microbiological indicators during treatment are poorly characterized, so when inadequate treatment is commenced, it often continues for months before incipient treatment failure is ascertained. Neither conventional tuberculosis microscopy nor PCR discriminate between live and dead tuberculosis bacilli, so these assays may have intrinsically limited reliability for predicting treatment failure, whether testing sputum or stool.^6,21^ Consequently, culture is the gold standard for identifying whether treatment is killing tuberculosis bacteria in vivo. However, culture is slow, so results provide out-of-date information. Therefore, finding tools for the early prediction of treatment failure is a priority. Viability microscopy and RNA-PCR have shown promise to monitor treatment response within 2 weeks of treatment initiation with same-day results, and the former can be used in microscopy centers with infrastructure that is already widely available.^22,23^ The novel technique for nucleic acid concentration that has been adapted from soil processing as evaluated by DiNardo et al may have important potential for more accurately assessing tuberculosis treatment response if it can be adapted to concentrate RNA from stool samples.^23^

In addition to its potential for predicting treatment failure, the improved sensitivity of stool PCR reported by DiNardo et al. has the potential to increase the laboratory confirmation of tuberculosis. This should decrease the number of cases with empirical treatment, increase the proportion of patients with tuberculosis who receive prompt and appropriate therapy, prevent people with other diagnoses receiving unnecessary tuberculosis therapy instead of the specific treatment that they actually need, and reduce the risk of inadvertent mistreatment of drug-resistant tuberculosis causing selection of drug resistance, treatment failure, death, and spread of tuberculosis to others.
